# Non-parametric correction of estimated gene trees using TRACTION

**DOI:** 10.1186/s13015-019-0161-8

**Published:** 2020-01-04

**Authors:** Sarah Christensen, Erin K. Molloy, Pranjal Vachaspati, Ananya Yammanuru, Tandy Warnow

**Affiliations:** Department of Computer Science, University of Illinois at Urbana-Champaign, Goodwin Ave, Urbana, IL USA

**Keywords:** Gene tree correction, Horizontal gene transfer, Incomplete lineage sorting

## Abstract

**Motivation:**

Estimated gene trees are often inaccurate, due to insufficient phylogenetic signal in the single gene alignment, among other causes. Gene tree correction aims to improve the accuracy of an estimated gene tree by using computational techniques along with auxiliary information, such as a reference species tree or sequencing data. However, gene trees and species trees can differ as a result of gene duplication and loss (GDL), incomplete lineage sorting (ILS), and other biological processes. Thus gene tree correction methods need to take estimation error as well as gene tree heterogeneity into account. Many prior gene tree correction methods have been developed for the case where GDL is present.

**Results:**

Here, we study the problem of gene tree correction where gene tree heterogeneity is instead due to ILS and/or HGT. We introduce TRACTION, a simple polynomial time method that provably finds an optimal solution to the RF-optimal tree refinement and completion (RF-OTRC) Problem, which seeks a refinement and completion of a singly-labeled gene tree with respect to a given singly-labeled species tree so as to minimize the Robinson−Foulds (RF) distance. Our extensive simulation study on 68,000 estimated gene trees shows that TRACTION matches or improves on the accuracy of well-established methods from the GDL literature when HGT and ILS are both present, and ties for best under the ILS-only conditions. Furthermore, TRACTION ties for fastest on these datasets. We also show that a naive generalization of the RF-OTRC problem to multi-labeled trees is possible, but can produce misleading results where gene tree heterogeneity is due to GDL.

## Background

Reconstructing the evolutionary history of a gene is a core task in phylogenetics, and our ability to infer these evolutionary relationships accurately can have important implications for a variety of downstream analyses. For example, estimated gene trees are used in the inference of adaptation, evolutionary event detection (such as gene loss, gene duplication, and horizontal gene transfer), ortholog identification, analysis of functional trait evolution, and species tree estimation. However, unlike species tree estimation techniques that leverage information encoded across the entire genome, gene tree estimation based on a single locus may not contain enough signal to determine the correct gene tree topology with high confidence [1]. Indeed, many phylogenomic datasets have gene trees with average branch support well below 75%, which is a common lower bound for branches to be considered reliable. For example, the Avian Phylogenomic Project [2] reported average branch support values below 30%, and many other studies (surveyed in [3]) have had similar challenges. Estimating gene and species trees is further complicated by biological processes such as gene duplication/loss (GDL), incomplete lineage sorting (ILS), and horizontal gene transfer (HGT), that create heterogeneous tree topologies across the genome [4]. HGT has long been known to cause problems for bacterial phylogenetics, and ILS by itself has emerged as a major issue in phylogenomics, affecting most, if not all, genome-scale datasets [5].

Because gene trees often have low accuracy, a natural problem is to try to improve gene tree estimation using an estimated or known species tree. An approach from the GDL literature is to modify estimated gene trees with respect to a reference species tree, which may either be an established tree from prior studies or an estimated species tree (e.g., based on an assembled multi-locus dataset). Some of these methods use the available sequence data as well as the estimated gene tree and species tree, and are referred to as *integrative methods*; examples include ProfileNJ [1], TreeFix [6], and TreeFix-DTL [7]. Other methods, called *gene tree correction methods*, use just the topologies of the gene tree and species tree, and are typically based on parametric models of gene evolution; Notung [8, 9] and ecceTERA [10] are two well-known methods of this type. Integrative methods are generally expected to be more accurate than gene tree correction methods when gene tree heterogeneity is due to GDL, but as a result of using likelihood calculations they are also more computationally intensive. See [10–16] for an entry into the vast literature on this subject.

Here, we examine gene tree correction where gene tree heterogeneity is due to ILS or HGT, and where each gene tree has at most one copy of each species. We present a new approach to gene tree correction that is based on a very simple *non-parametric* polynomial-time method, TRACTION. In addition to correcting gene trees, TRACTION is also capable of completing gene trees that do not contain all the species present in the reference species tree, a condition that may occur in a multi-locus study when not all genomes have been sequenced and assembled.

The input to TRACTION is a pair (*t*, *T*) of unrooted, singly-labeled phylogenetic trees. The leaf set of *t* is a subset of the leaf set of *T*, tree *T* is binary, and tree *t* will generally be non-binary. We seek a tree $T'$ created by refining *t* and adding any missing leaves so that $T'$ has the minimum Robinson−Foulds (RF) [17] distance to *T*. We call this the *RF-optimal tree refinement and completion Problem* (RF-OTRC) and show that TRACTION finds an optimal solution to RF-OTRC in $O(n^{1.5} \log n)$ time, where *n* is the number of leaves in the species tree *T*. We also explore an extension of this problem statement to handle multi-labeled genes by using a generalization of the RF distance proposed in [18].

To use TRACTION for gene tree correction in practice, we assume we are given an estimated gene tree with branch support values and an estimated (or known) binary species tree, which may have additional species. The low support branches in the gene tree are collapsed, forming the (unresolved) tree *t*. TRACTION first refines the input gene tree *t* into a binary tree $t'$, and then it adds the missing species to $t'$. Although the algorithm is quite simple, the proof of correctness is non-trivial.

We present the results of an extensive simulation study (on 68,000 gene trees, each with up to 51 species) in which gene tree heterogeneity is either due to only ILS or to both ILS and HGT. We explore TRACTION for gene tree correction with estimated species trees in comparison to Notung, ecceTERA, ProfileNJ, TreeFix, and TreeFix-DTL. Many methods (including TRACTION) tie for best on the ILS-only data, but TRACTION dominates the other gene tree correction methods with respect to topological accuracy on the HGT + ILS data, while also tying for fastest. Importantly, TRACTION provides good accuracy even when the estimated species tree is far from the true gene tree. The simplicity of the approach and its good accuracy under a range of model conditions indicate that non-parametric approaches to gene tree correction may be promising and encourages future research.

## TRACTION

### Terminology and basics

A *phylogenetic tree* can be represented as a tree *T* with leaves labeled by some set of organisms *S*. If each leaf label is unique, then the phylogenetic tree is *singly-labeled*. Unless noted otherwise, the phylogenetic trees we describe throughout this paper are singly-labeled and unrooted.

Each edge *e* in an unrooted, singly-labeled phylogenetic tree defines a *bipartition* $\pi _e$ (also sometimes referred to as a split) on the set of leaf labels induced by the deletion of *e* from the tree, but not its endpoints. Each bipartition splits the leaf set into two non-empty disjoint parts, *A* and *B*, and is denoted by *A*|*B*. The set of bipartitions of a tree *T* is given by *C*(*T*) = {$\pi _e$ : $e \in E(T)$}, where *E*(*T*) is the edge set for *T*. Tree $T'$ is a *refinement* of *T* if *T* can be obtained from $T'$ by contracting a set of edges in $E(T')$. A tree *T* is *fully resolved* (i.e., binary) if there is no tree that refines *T* other than itself.

A set *Y* of bipartitions on some leaf set *S* is *compatible* if there exists an unrooted tree *T* leaf-labeled by *S* such that *Y* $\subseteq$ *C*(*T*). A bipartition $\pi$ of a set *S* is said to be compatible with a tree *T* with leaf set *S* if and only if there is a tree $T'$ such that $C(T') = C(T) \cup \{\pi \}$ (i.e., $T'$ is a refinement of *T* that includes the bipartition $\pi$). Similarly, two trees on the same leaf set are said to be compatible if they share a common refinement. An important result on compatibility is that pairwise compatibility of a set of bipartitions over a leaf set ensures setwise compatibility [19, 20]; it then follows that two trees are compatible if and only if the union of their sets of bipartitions is compatible. Furthermore, by [21] (and see discussion in [22, 23]), a set $\mathcal {C}$ of bipartitions is compatible if and only if there is a tree *T* such that $C(T)=\mathcal {C}.$

The *Robinson−Foulds* (RF) distance [17] between two trees *T* and $T'$ on the same set of leaves is defined as the minimum number of edge-contractions and refinements required to transform *T* into $T'$ (where each such operation changes the number of edges in the tree by exactly one, so contracting a single edge or refining a polytomy to add a single edge). For singly-labeled trees, the RF distance equals the number of bipartitions present in only one tree (i.e., the symmetric difference). The normalized RF distance is the RF distance divided by $2n-6$, where *n* is the number of leaves in each tree; this produces a value between 0 and 1 since the two trees can only disagree with respect to internal edges, and $n-3$ is the maximum number of internal edges in an unrooted tree with *n* leaves.

**Fig. 1:**
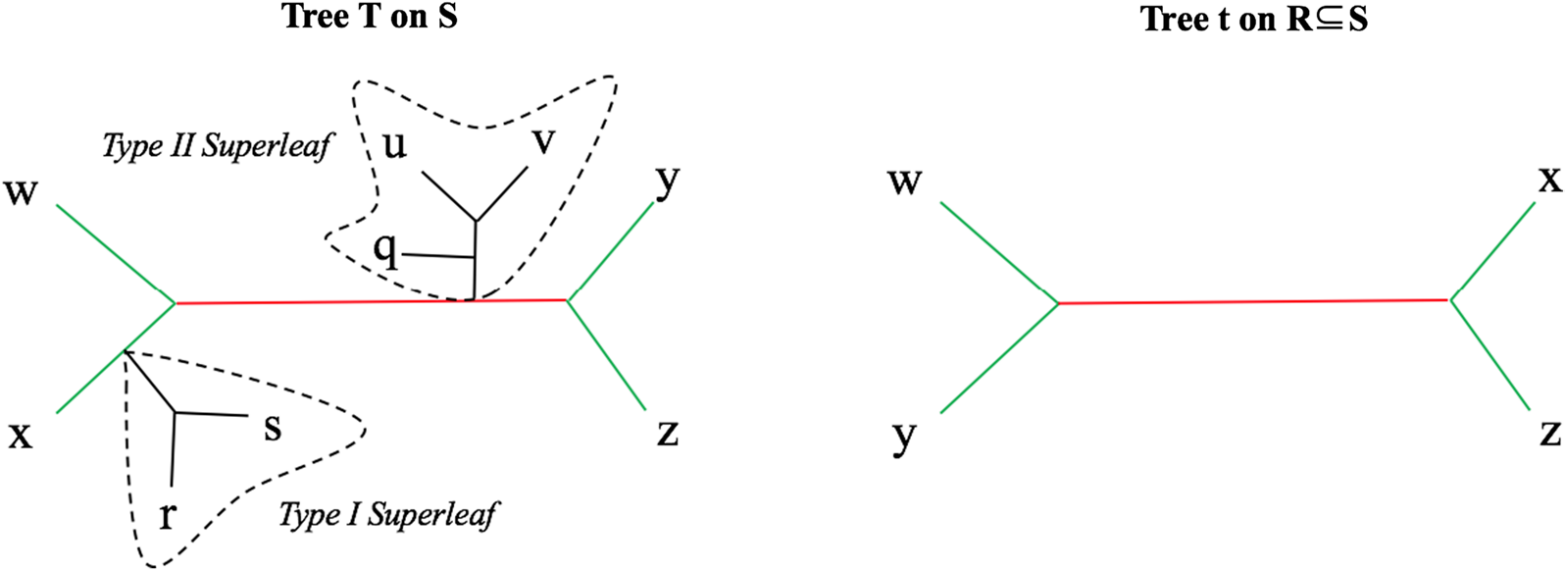
Type I and Type II superleaves of a tree *T* with respect to *t*. Edges in the backbone (defined to be the edges on paths between nodes in the common leaf set) are colored green for shared, red for unique; all other edges are colored black. The deletion of the backbone edges in *T* defines the superleaves; one is a Type I superleaf because it is attached to a shared (green) edge and the other is a Type II superleaf because it is attached to a unique (red) edge. This figure is from [25], reused under the Creative Commons Attribution (CC-BY) license

Given a phylogenetic tree *T* on taxon set *S*, *T* *restricted to* $R \subseteq S$ is the minimal subgraph of *T* connecting elements of *R* and suppressing nodes of degree two. We denote this as $T|_R$. If *T* and $T'$ are two trees with *R* as the intersection of their leaf sets, their *shared edges* are edges whose bipartitions restricted to *R* are in the set $C(T|_R)\cap C(T'|_R)$. Correspondingly, their *unique edges* are edges whose bipartitions restricted to *R* are not in the set $C(T|_R)\cap C(T'|_R)$. See Fig. 1 for a pictorial depiction of unique and shared edges.

### RF-optimal tree refinement and completion (RF-OTRC) problem

We now turn our attention to the optimization problem of interest to this paper. This section is limited to the context of singly-labeled trees; we postpone the extension to cases where the gene tree can have multiple copies of a species at the leaves, which are referred to as multi-labeled trees (i.e., MUL-trees [24]), until a later section.
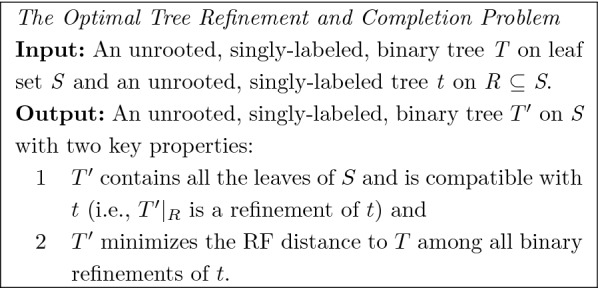


If the trees *t* and *T* have the same set of taxa, then the RF-OTRC problem becomes the RF-optimal tree refinement (RF-OTR) problem, while if *t* is already binary but can be missing taxa, then the RF-OTRC problem becomes the RF-optimal tree completion (RF-OTC) problem. OCTAL, presented in [25], solves the RF-OTC problem in $O(n^2)$ time, and an improved approach presented by Bansal [26] solves the RF-OTC problem in linear time. We refer to this faster approach as *Bansal’s algorithm*. In this paper we present an algorithm that solves the RF-OTR problem exactly in polynomial time and show that the combination of this algorithm with Bansal’s algorithm solves the RF-OTRC problem exactly in $O(n^{1.5} \log n)$ time, where *T* has *n* leaves. We refer to the two steps together as Tree Refinement And CompleTION (TRACTION).

### TRACTION algorithm

The input to TRACTION is a pair of unrooted, singly-labeled trees (*t*, *T*), where *t* is the estimated gene tree on set *R* of species and *T* is the binary reference tree on *S*, with $R \subseteq S$. Note that we allow *t* to not be binary (e.g., if low support edges have already been collapsed) and to be missing species (i.e., $R \subset S$ is possible).

- **Step 1**: Refine *t* so as to produce a binary tree $t^*$ that maximizes shared bipartitions with *T*.
- **Step 2**: Add the missing species from *T* into $t^*$, minimizing the RF distance.

#### Step 1: Greedy refinement of *t*

To compute $t^*$, we first refine *t* by adding all bipartitions from $T|_{R}$ that are compatible with *t*; this produces a unique tree $t'$. If $t'$ is not fully resolved, then there are multiple optimal solutions to the RF-OTR problem, as we will later prove. The algorithm selects one of these optimal solution as follows. First, we add edges from *t* that were previously collapsed (if such edges are available). Next, we randomly refine the tree until we obtain a fully resolved refinement, $t^*$. Note that if $t'$ is not binary, then $t^*$ is not unique. We now show that the first step of TRACTION solves the RF-OTR problem.

##### **Theorem 1**

*Let T be an unrooted, singly-labeled tree on leaf set S, and let t be an unrooted, singly-labeled tree on leaf set* $R \subseteq S$*. A fully resolved (i.e. binary) refinement of t minimizes the RF distance to* $T|_{R}$* if and only if it includes all compatible bipartitions from* $T|_{R}$.

##### *Proof*

Let $C_0$ denote the set of bipartitions in $T|_R$ that are compatible with *t*. By the theoretical properties of compatible bipartitions (see “Terminology and basics” section), this means the set $C_0 \cup C(t)$ is a compatible set of bipartitions that define a unique tree $t'$ where $C(t')=C_0 \cup C(t)$ (since the trees are singly-labeled).

We now prove that for any binary tree *B* refining *t*, *B* minimizes the RF distance to $T|_R$ if and only if *B* refines $t'$.

Consider a sequence of trees $t=t_0, t_1, t_2, \ldots , t_k$, each on leaf set *R*, where $t_i$ is obtained from $t_{i-1}$ by adding one edge to $t_{i-1}$, and thus adds one bipartition to $C(t_{i-1})$. Let $\delta _i=RF(t_{i},T|_R) - RF(t_{i-1},T|_R)$, so that $\delta _i$ indicates the change in RF distance produced by adding a specific edge to $t_{i-1}$ to get $t_i$. Hence,

$$\begin{aligned} RF(t_i,T|_R) = RF(t_0,T|_R) + \sum _{j \le i} \delta _j. \end{aligned}$$

A new bipartition $\pi _i$ added to $C(t_{i-1})$ is in $C(T|_R)$ if and only if $\pi _i \in C_0$. If this is the case, then the RF distance will decrease by one (i.e., $\delta _i =-1$). Otherwise, $\pi _i \not \in C_0$, and the RF distance to $T|_R$ will increase by one (i.e., $\delta _i =1$).

Now suppose *B* is a binary refinement of *t*. We can write the bipartitions in $C(B){\backslash}C(t)$ into two sets, *X* and *Y*, where *X* are bipartitions in $C_0$ and *Y* are bipartitions not in $C_0$. By the argument just provided, it follows that $RF(B,T|_R) = RF(t,T|_R) - |X| + |Y|$. Note that $|X \cup Y|$ must be the same for all binary refinements of *t*, because all binary refinements of *t* have the same number of edges. Thus, $RF(B,T|_R)$ is minimized when |*X*| is maximized, so *B* minimizes the RF distance to $T|_R$ if and only if *C*(*B*) contains all the bipartitions in $C_0$. In other words, $RF(B,T|_R)$ is minimized if and only if *B* refines $t'$. $\square$

##### **Corollary 1**

*TRACTION finds an optimal solution to the RF-OTR problem.*

##### *Proof*

Given input gene tree *t* and reference tree *T* on the same leaf set, TRACTION produces a tree $t''$ that refines *t* and contains every bipartition in *T* compatible with *t*; hence by Theorem 1, TRACTION solves the RF-OTR problem. $\square$

#### Step 2: Adding in missing species

The second step of TRACTION can be performed using OCTAL or Bansal’s algorithm, each of which finds an optimal solution to the RF-OTC problem in polynomial time. Indeed, we show that any method that optimally solves the RF-OTC problem can be used as an intermediate step to solve the RF-OTRC problem.

To prove this, we first restate several prior theoretical results. In [25] we showed the minimum achievable RF distance between *T* and $T'$ is given by:

1 $$\begin{aligned} RF(T,T')&= RF(T|_R,t) +2m \end{aligned}$$

where *m* is the number of Type II superleaves in *T* relative to *t*, which we define:

##### **Definition 1**

Let *T* be a binary tree on leaf set *S* and *t* be a tree on leaf set $R \subseteq S$. The *superleaves* of *T* with respect to *t* are defined as follows (see Fig. 1). The set of edges in *T* that are on a path between two leaves in *R* define the *backbone*; when this backbone is removed, the remainder of *T* breaks into pieces. The components of this graph that contain vertices from $S \setminus R$ are the superleaves. Each superleaf is rooted at the node that was incident to one of the edges in the backbone, and is one of two types:

- *Type I superleaves*: the edge *e* in the backbone to which the superleaf was attached is a shared edge in $T|_R$ and *t*
- *Type II superleaves*: the edge *e* in the backbone to which the superleaf was attached is a unique edge in $T|_R$ and *t*

##### **Theorem 2**

(Restatement of Theorem 9 in [25])* Given unrooted, singly-labeled binary trees t and 7 with the leaf set of t a subset of the leaf set S of T, OCTAL(T, t) solves the RF-OTC problem and runs in *$O(n^2)$ *time, where T has n leaves.*

### Proof of correctness for TRACTION

#### **Lemma 1**

*Let T be an unrooted, singly-labeled, binary tree on leaf set S with *$|S|=n$*, and let t be an unrooted, singly-labeled tree on leaf set *$R \subseteq S$*. TRACTION returns a binary unrooted tree *$T'$ *on leaf set S such that *$RF(T',T)$ *is minimized subject to *$T'|_{R}$ *refining t*.

#### *Proof*

By construction *TRACTION* outputs a tree $T'$ that, when restricted to the leaf set of *t*, is a refinement of *t*. Hence, it is clear that $T'|_{R}$ refines *t*. Now, it is only necessary to prove that RF($T'$, *T*) is minimized by *TRACTION*. Since the intermediate tree $t^*$ produced in the first step of TRACTION is binary, Theorem 2 gives that TRACTION using OCTAL (or any method exactly solving the RF-OTC problem) will add leaves to $t^*$ in such a way as to minimize the RF distance to *T*; hence it suffices to show that $t^*$ computed by TRACTION has the smallest RF distance to *T* among all binary refinements of *t*.

As given in Eq. 1, the optimal RF distance between $T'$ and *T* is the sum of two terms: (1) RF($t^*$, $T|_R$) and (2) the number of Type II superleaves in *T* relative to $t^*$. Theorem 1 shows that TRACTION produces a refinement $t^*$ that minimizes the first term. All that remains to be shown is that $t^*$ is a binary refinement of *t* minimizing the number of Type II superleaves in *T* relative to $t^*$.

Consider a superleaf *X* in *T* with respect to *t*. If *t* were already binary, then every superleaf *X* is either a Type I or a Type II superleaf. Also, note that every Type I superleaf in *T* with respect to *t* will be a Type I superleaf for any refinement of *t*. However, when *t* is not binary, it is possible for a superleaf *X* in *T* to be a Type II superleaf with respect to *t* but a Type I superleaf with respect to a refinement of *t*. This happens when the refinement of *t* introduces a new shared edge with *T* to which the superleaf X is attached in *T*. Notice that since the set of all possible shared edges that could be created by refining *t* is compatible, any refinement that maximizes the number of shared edges with *T* also minimizes the number of Type II superleaves. Theorem 1 shows that *TRACTION* produces such a refinement $t^*$ of *t*. Thus, *TRACTION* finds a binary unrooted tree $T'$ on leaf set *S* such that RF($T'$, *T*) is minimized subject to the requirement that $T'|_{R}$ refine *t*. $\square$

#### **Theorem 3**

*TRACTION solves the RF-OTRC problem and runs in* $O(n^{1.5}\log n)$* time if used with Bansal’s algorithm and* $O(n^2)$*time if used with OCTAL, where n is the number of leaves in the species tree.*

#### *Proof*

The above lemma shows that TRACTION solves the RF-OTRC problem. Let *t*, *T*, *S*, and *R* be as defined in the RF-OTRC problem statement. What remains to be shown is a running time analysis for the first stage of TRACTION (refining *t*). We claim this step takes $O(|S|+ |R|^{1.5} \log (|R|))$ time.

Constructing $T|_R$ takes *O*(|*S*|) time. Checking compatibility of a single bipartition with a tree on *K* leaves, and then adding the bipartition to the tree if compatible, can be performed in only $O(|K|^{0.5} \log (|K|))$ after a fast preprocessing step (see Lemmas 3 and 4 from [27]). Hence, determining the set of edges of $T|_R$ that are compatible with *t* takes only $O(|S| + |R|^{1.5} \log (|R|))$ time. Therefore, the first stage of TRACTION takes $O(|S|+ |R|^{1.5} \log (|R|))$ time. Hence, if used with OCTAL, TRACTION takes $O(|S|^{2})$ time and if used with Bansal’s algorithm TRACTION takes $O(|S|^{1.5} \log |S|)$ time. $\square$

### Extending TRACTION to MUL-trees

Up to this point, we have formulated gene tree correction problems only in the context where the input trees are each singly-labeled (i.e., have at most one leaf for each species). However, in the context of GDL, a gene tree may have multiple copies of a species at its leaves (i.e., it can be a “MUL-tree”). We now generalize the RF-OTR problem to allow the input unresolved tree *t* to be a MUL-tree, although we still require the species tree *T* to be singly-labeled.

Recall that the RF distance between two trees is the minimum number of contractions and refinements that suffice to transform one tree into the other, and that this is equal to the bipartition distance for singly-labeled trees. This definition requires that the two trees have the same number of copies of each species (also referred to as “label-multiplicity”), since otherwise there is no such edit transformation. However, even when the two MUL-trees have the same number of copies of each species, we cannot rely on the use of the bipartition distance, as two MUL-trees can have identical sets of bipartitions but not be isomorphic [28].

In the context we will address, we are given a MUL-tree $\mathcal {R}$ (i.e., the gene family tree) and a singly-labeled tree *T* (i.e., the species tree). To extend the RF-OTR problem so that we can use it for such an input pair, we will draw on some definitions and results from [11, 28].

#### **Definition 2**

Let *r* and *t* be given with *r* a MUL-tree and *t* a singly-labeled tree, and both with the same set of species labeling the leaves. We construct the MUL-tree *Ext*(*t*, *r*) from *t* as follows: for each species *s* and the unique leaf *x* in *t* labeled by *s*, we replace *x* by a node $v_s$ that is attached to *k* leaves, each labeled by *s*, where *k* is the number of leaves in *r* that are labeled by *s*. We refer to *Ext*(*t*, *r*) as the **extension of** *t* **relative to** *r*. Note that *Ext*(*t*, *r*) and *r* have the same number of copies of each species.
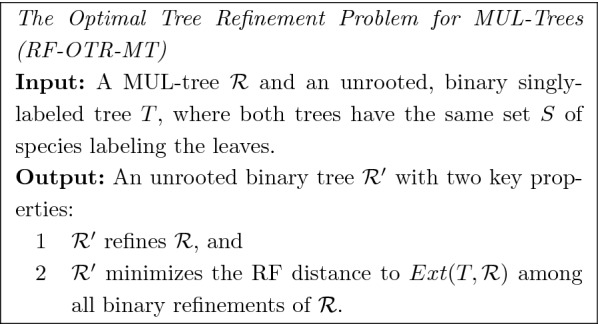


Before we present TRACTION-MT (i.e., TRACTION for MUL-trees), we need one more definition.

#### **Definition 3**

Let $r_1$ and $r_2$ be MUL-trees, both leaf-labeled by the same set of species, with the same number of copies of each species labeling the leaves. We construct $r_1'$ from $r_1$ (and similarly $r_2'$ from $r_2$) by relabeling the leaves of $r_1$ so that it is singly-labeled by replacing the *k* leaves labeled by *s* with $s_1, s_2, \ldots , s_k$. Note that $r_1'$ and $r_2'$ are now singly-labeled trees and that $L(r_1')=L(r_2')$. We say the pair $(r_1',r_2')$ is a **consistent full differentiation** of $(r_1,r_2)$.

We now present *TRACTION-MT*. The input to TRACTION-MT is a pair $(\mathcal {R},T)$ where $\mathcal {R}$ is a MUL-tree and *T* is a singly-labeled tree, and they are both leaf-labeled by a set *S* of species.

- Step 1: Compute $Ext(T,\mathcal {R})$ (i.e., the extended version of *T* with respect to $\mathcal {R}$, see Definition 2).
- Step 2: Relabel the leaves in *T* and $Ext(T,\mathcal {R})$ in a mutually consistent fashion (see Definition 3), thus producing trees $T'$ and $\mathcal {R}'$.
- Step 3: Apply TRACTION to the pair $$\mathcal {R}'$$ and $$T'$$, producing tree $$\mathcal {R}^*$$ on leafset $$S'$$. For every species $$s \in S$$ and leaf in $$\mathcal {R}^*$$ labeled $$s_i$$, replace the label $$s_i$$ by *s*, thus producing a tree $$\mathcal {R}^{**}$$ on leaf-set *S* that is isomorphic to $$\mathcal {R}^*$$.
- Step 4: Return $$\mathcal {R}^{**}$$.

#### **Theorem 4**

*TRACTION-MT solves the RF-OTR-MT problem exactly and has running time* $O(|\mathcal {R}|^{1.5} \log |\mathcal {R}|)$.

#### *Proof*

Let MUL-tree $$\mathcal {R}$$ and singly-labeled tree *T* be given, and let $$\mathcal {R}^{**}$$ be the tree returned by TRACTION-MT for this pair. We will show that $$\mathcal {R}^{**}$$ is a refinement of $$\mathcal {R}$$ that has minimum RF distance to $$Ext(T,\mathcal {R})$$ among all binary refinements, thus establishing that TRACTION-MT solves the RF-OTR-MT problem optimally [28].

Steps 1 and 2 together take the input pair $$\mathcal {R}$$ and *T* and creates two new trees $$\mathcal {R}'$$ and $$T'$$ that form a pair of consistent full differentiations of $$\mathcal {R}$$ and $$Ext(T,\mathcal {R})$$. By Theorem 3 in [11], $$RF(\mathcal {R},Ext(T,\mathcal {R})) = RF(\mathcal {R}',T')$$. Since $$\mathcal {R}'$$ and $$T'$$ are singly-labeled, Step 2 produces a tree $$\mathcal {R}^*$$ that is a refinement of $$\mathcal {R}'$$ and minimizes the RF distance to $$T'$$. Therefore the tree $$\mathcal {R}^{**}$$ is a refinement of $$\mathcal {R}$$ that minimizes the RF distance to $$Ext(T,\mathcal {R})$$. Hence, TRACTION-MT finds an optimal solution to the RF-OTR-MT problem on this input pair.

Finally, for the running time analysis, the creation of the two trees $\mathcal {R}'$ and $\mathcal {T}'$ takes $O(|\mathcal {R}|)$. Then running TRACTION on this pair takes an additional $O(|\mathcal {R}|^{1.5} \log |\mathcal {R}|)$ time, as noted in Theorem 3. $\square$

**Fig. 2:**
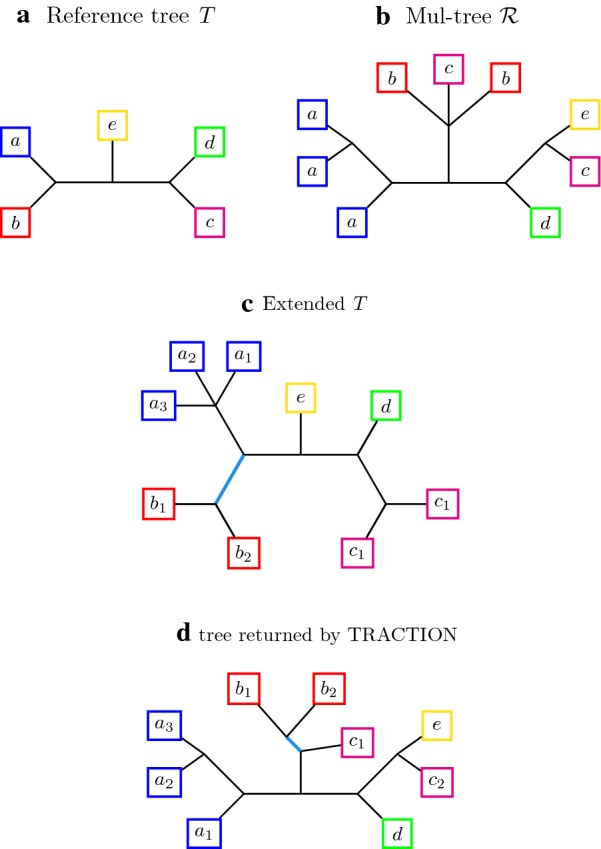
Example of MUL-tree correction using TRACTION-MT given a reference tree. Given a singly-labeled, binary tree *T* on leaf set *S*, we wish to correct a MUL-tree $\mathcal {R}$ using TRACTION-MT. First, we build the extension of *T* with respect to $\mathcal {R}$, called “Extended *T*.” Second, we re-label the leaves so that $\mathcal {R}$ and Extended *T* become consistent full differentiations. Now we run TRACTION on the pair, producing the singly-labeled tree shown in (d). TRACTION-MT would then relabel the leaves again (i.e., $s_i$ is relabeled *s* for all species *s*), to produce a MUL-tree that refines $\mathcal {R}$

Figure 2 provides example of a MUL-tree, an extended species tree, and TRACTION’s solution to the RF-OTR problem for MUL-trees.

## Evaluation

### TRACTION-MT under gene duplication and loss: case study

**Fig. 3:**
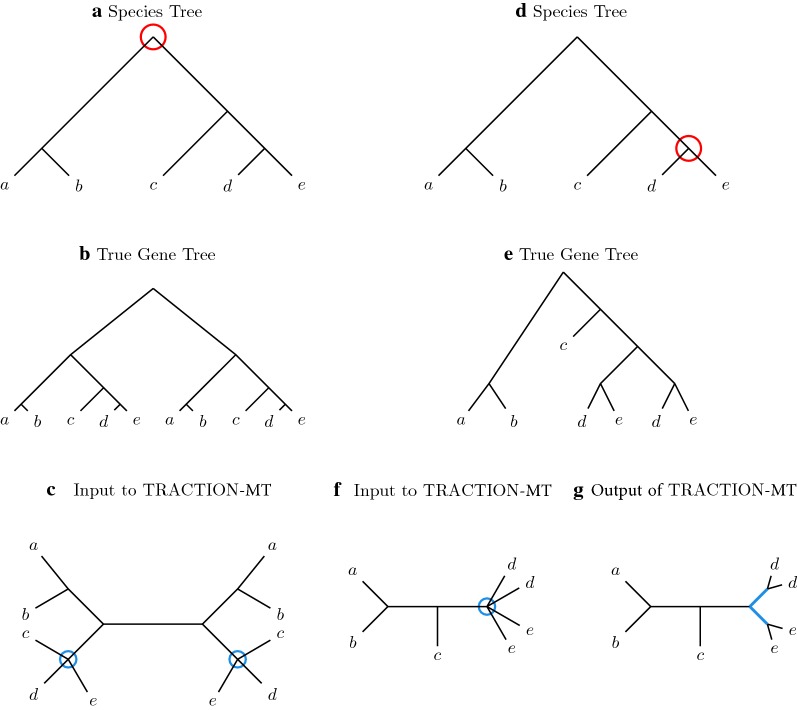
Two cases where TRACTION-MT does not have good accuracy on multi-labeled gene trees. In the first case (left column), a duplication event (red circle) occurs at the root of the species tree shown in **a**, producing the true gene tree shown in **b**. If TRACTION-MT is given the estimated gene tree shown in **c** and the unrooted true species tree (**a**) as input, then TRACTION-MT will randomly refine the estimated gene tree, because it cannot add any bipartitions from the species tree. In the second case (right column), a duplication event (red circle) occurs towards the leaves of the species tree shown in **d**, producing the true gene tree shown in **e**. If TRACTION-MT is given the estimated gene tree shown in **f** and the unrooted true species tree (**d**) as input, then TRACTION-MT will add two branches as shown in blue in **g**, producing an incorrect gene tree. Furthermore, the addition of these two incorrect branches would imply two duplication events, one occurring at leaf *d* and one occurring at leaf *e*, in the true species tree, so that the gene tree returned by TRACTION-MT will not minimize the number of duplication events.

There are model conditions under which TRACTION-MT will not accurately modify an input estimated gene tree, even when given the true species tree as the reference tree and a collapsed version of the true gene tree. For example, if a duplication event takes place at the root of a species tree, then genes of the same species will not be siblings in the true gene tree. Hence, if TRACTION-MT is given the true gene tree (i.e., MUL-tree), it will not be able to add any bipartitions to it from the extended species tree, and will instead return a random refinement (see Fig. 3a–c). For a second example, if a duplication event takes place closer to the leaves, then genes of the same species appear somewhat close to each other in the true gene tree. As a result, TRACTION-MT may add edges in the wrong place, resulting in incorrect locations for duplications (see Fig. 3d–g). The key point to both cases is that when TRACTION-MT adds edges from the extended species tree, these imply duplications at the leaves of the species tree, and the edges produced by random refinements of the MUL-tree have low probability (i.e., never more than $\frac{1}{3}$) of being in the true species tree.

### TRACTION under ILS and HGT: simulations

#### Overview

We evaluated TRACTION in comparison to Notung, ecceTERA, ProfileNJ, TreeFix, and TreeFix-DTL on estimated gene trees under two different model conditions (ILS-only and ILS+HGT), using estimated and true species trees. In total, we analyzed 68,000 genes: 8000 with 26 species under ILS-only models and 60,000 with 51 species under ILS + HGT models. All estimated gene trees that we correct in these experiments were complete (i.e., were not missing species). The motivation for this is twofold. First, the methods we benchmarked against do not provide an option for completing gene trees with missing data. This is understandable since these methods were developed for GDL, where missing species in a gene tree are interpreted as true loss events rather than incomplete sampling. Second, an experimental evaluation of OCTAL, the algorithm that performs the completion step of TRACTION, was previously performed in [25].

#### Datasets

We briefly describe the datasets used in this study; all datasets are from prior studies [25, 29] and available online. The datasets included singly-labeled genes with 26 or 51 species (each with a known outgroup), and were generated under model conditions where true gene trees and true species trees differed due to only ILS (datasets with 26 species had two levels of ILS) or due to both ILS and HGT (datasets with 51 species had the same level of ILS but two different levels of HGT). The true gene tree heterogeneity (*GT-HET*, the topological distance between true species trees and true gene trees) ranged from 10% (for the ILS-only condition with moderate ILS) to as high as 68% (for the ILS+HGT condition with high HGT). Each model condition has 200 genes, and we explored multiple replicate datasets per model condition with different sequence lengths per gene. See Table 1 for details.

**Table 1:** Empirical properties of the simulated datasets used in this study: gene tree heterogeneity, the average normalized RF distance between true gene trees and true species trees (GT-HET); average gene tree estimation error (GTEE); and the average distance of the ASTRID reference tree, to the true gene trees

	GT-HET	GTEE	Distance ASTRID to true gene trees
ILS-only, low ILS, 26 species [25]			
# sites varies	0.10	0.32	0.08
ILS-only, high ILS, 26 species [25]			
# sites varies	0.36	0.40	0.33
ILS+HGT, moderate HGT (m5), 51 species [29]			
100 sites	0.54	0.63	0.55
250 sites	0.54	0.47	0.55
500 sites	0.54	0.47	0.54
ILS+HGT, high HGT (m6), 51 species [29]			
100 sites	0.68	0.62	0.68
250 sites	0.68	0.46	0.68
500 sites	0.68	0.38	0.68

The publications from which the simulated datasets are taken are also indicated. In total we analyzed 68,000 genes with varying levels and causes of true gene tree heterogeneity (to the true species tree) and gene tree estimation error. The ILS-only conditions each had 20 replicates, and the ILS+HGT conditions each had 50 replicates

#### Estimated gene trees and estimated reference species trees

For each gene, we used RAxML v8.2.11 [30] under the GTRGAMMA model to produce maximum likelihood gene trees, with branch support computed using bootstrapping. Because sequence lengths varied, this produced estimated gene trees with different levels of gene tree estimation error *(GTEE)* (defined to be the average RF distance between the true gene tree and the estimated gene tree), ranging from 32 to 63% as defined by the missing branch rate (see Table 1). We estimated a species tree using ASTRID v1.4 [31] given the RAxML gene trees as input. Because the true outgroup for all species trees and gene trees was known, we rooted the species tree and all gene trees at the outgroup prior to performing gene tree correction.

The gene trees given as input to the different correction methods were computed as follows. Each gene tree estimated by RAxML had branches annotated with its bootstrap support, and we identified all the branches with bootstrap support less than a given threshold. These branches with low support were then collapsed in the gene trees before being given to TRACTION, Notung, and ProfileNJ. When we ran ecceTERA, we gave the binary gene trees with the threshold value (i.e., minimum required bootstrap support value); ecceTERA collapses all branches that have support less than the threshold value, and explores the set of refinements. Thus, the protocol we followed ensured that ecceTERA, ProfileNJ, Notung, and TRACTION all used the same set of collapsed gene trees. TreeFix and Treefix-DTL used the uncollapsed gene trees. We ran all methods using a threshold value of 75% (the standard threshold for “low support”). We additionally ran TRACTION and Notung using collapse thresholds of 50%, 85%, and 90% on the ILS-only data.

#### Gene tree correction and integrative methods

The RAxML gene trees were corrected using TRACTION v1.0, Notung v2.9, ecceTERA v1.2.4, ProfileNJ (as retrieved from GitHub after the March 20, 2018 commit with ID 560b8b2) [1], TreeFix v1.1.10 (for the ILS-only datasets), and TreeFix-DTL v1.0.2 (for the HGT + ILS datasets), each with a species tree estimated using ASTRID v1.4 [31] as the reference tree rooted at the outgroup. The integrative methods (TreeFix, TreeFix-DTL, and ProfileNJ) also required additional input data related to the gene alignments, which we detail in the commands below. All estimated gene trees were complete (i.e., there were no missing taxa), so TRACTION only refined the estimated gene tree and did not add any taxa. We also explored using the true model species tree as a reference tree for TRACTION and Notung on the ILS-only datasets.

#### Evaluation criteria

We used RF tree error (the standard criterion in performance studies evaluating phylogeny estimation methods) to quantify error in estimated and corrected gene trees as compared to the known true gene tree (as defined in the simulation protocol) and the impact of TRACTION, Notung, ecceTERA, and TreeFix-DTL, on these errors. Note that although we used the RF distance within the OTR optimization criterion, in that context, it refers to the distance between the corrected gene tree and the reference tree (which is an *estimated species tree*); in contrast, when we used the RF error rate in the evaluation criterion, it refers to the distance between the corrected gene tree and the *true gene tree*. Since the reference trees used in our experiments are typically very topologically different from the true gene tree (8% RF distance for the moderate ILS condition, 33% for the high ILS condition, 54% to 68% for the ILS+HGT conditions, see Table 1), optimizing the RF distance to the reference tree is quite different from optimizing the RF distance to the true gene tree. Finally, we also evaluated the methods using the matching distance [32] and the quartet distance [33].

#### Experiments

We performed two main experiments: one in which we explored performance on ILS-only datasets and the other in which we explored performance on datasets with HGT and ILS. In each case, we directly explored how the GTEE level impacted absolute and relative accuracy of gene tree correction methods. We also indirectly explored how GT-HET affects relative and absolute accuracy. Heterogeneity is higher on the HGT + ILS datasets than on the ILS-only datasets, as HGT adds heterogeneity between gene trees and species trees (see Table 1). In our third experiment, we evaluated how the branch support collapse threshold and how using the true species tree as the reference tree impacted absolute and relative performance among the best performing methods on the ILS-only datasets.

#### Commands

In the following commands, *resolved gene trees* refers to the gene trees estimated using RAxML, *unresolved gene trees* refers to these estimated gene trees with branches having bootstrap support less than the threshold (e.g., 75%) collapsed, and *reference species tree* refers to the species tree estimated using ASTRID. *Rooted* means the input tree was rooted at the outgroup.

RAxML v8.2.11 was run as 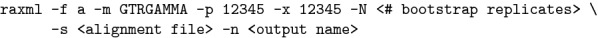


ASTRID v1.4 was run as 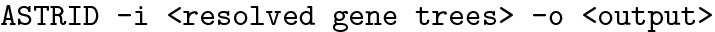


Notung v2.9 was run as 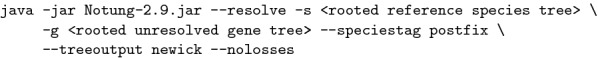


TRACTION v1.0 was run as 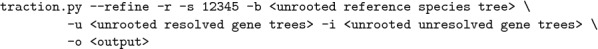


ecceTERA v1.2.4 was run as 
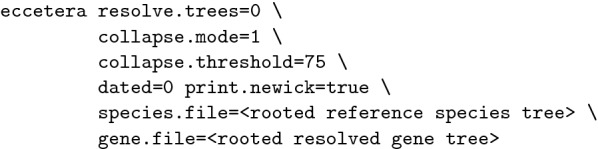


FastME v2.1.6.1 [34], used to compute a distance matrix for ProfileNJ, was run as 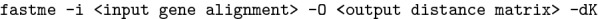


ProfileNJ, using the K2P-corrected distance matrix from FastME, was run as 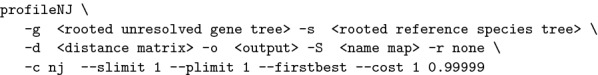


TreeFix v1.1.10 was run on the ILS-only datasets as 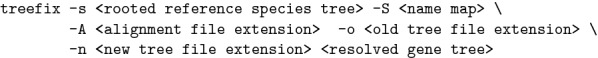


TreeFix-DTL v1.0.2 was run on the HGT + ILS datasets as 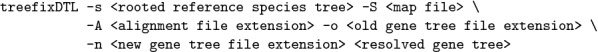


Normalized RF distances were computed using Dendropy v4.2.0 [35] as 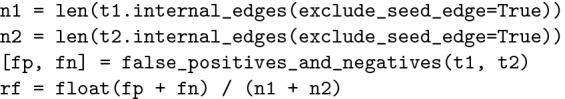


Matching distances were computed using code from [32] and [36] as 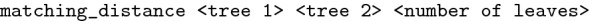


Quartet distances were computed using QDist [33] as 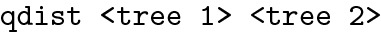


## Results and discussion

### Experiment 1: Comparison of methods on ILS-only datasets

**Fig. 4:**
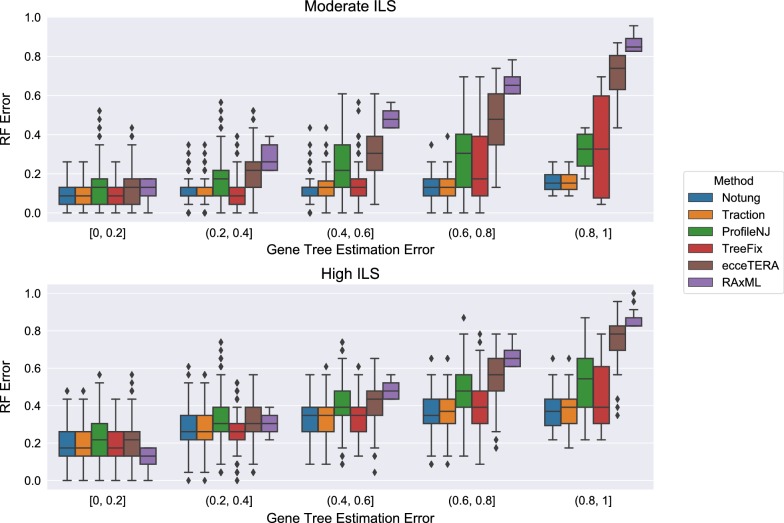
Comparison of methods on the ILS-only datasets with respect to Robinson−Foulds (RF) error rates as a function of GTEE. Results are only shown for those datasets on which all methods completed. Each model condition (characterized by ILS level) has 20 replicate datasets, each with 200 genes

**Fig. 5:**
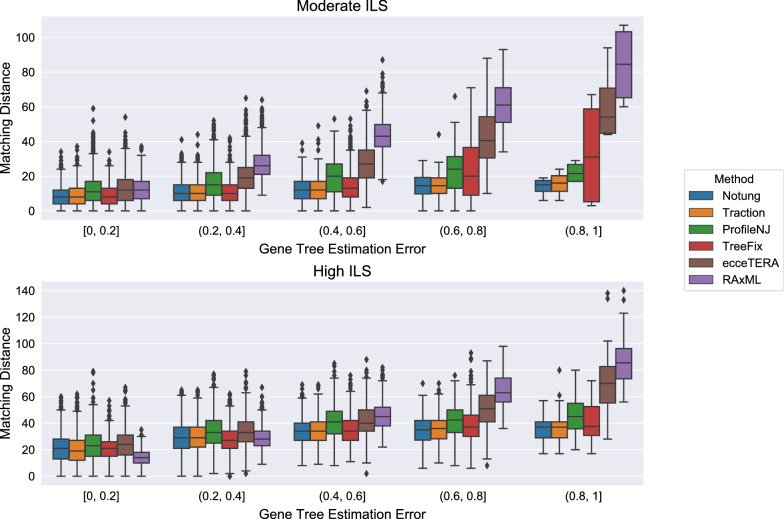
Comparison of methods on the ILS-only datasets with respect to matching distance as a function of GTEE. Results are only shown for those datasets on which all methods completed. Each model condition (characterized by ILS level) has 20 replicate datasets, each with 200 genes

**Fig. 6:**
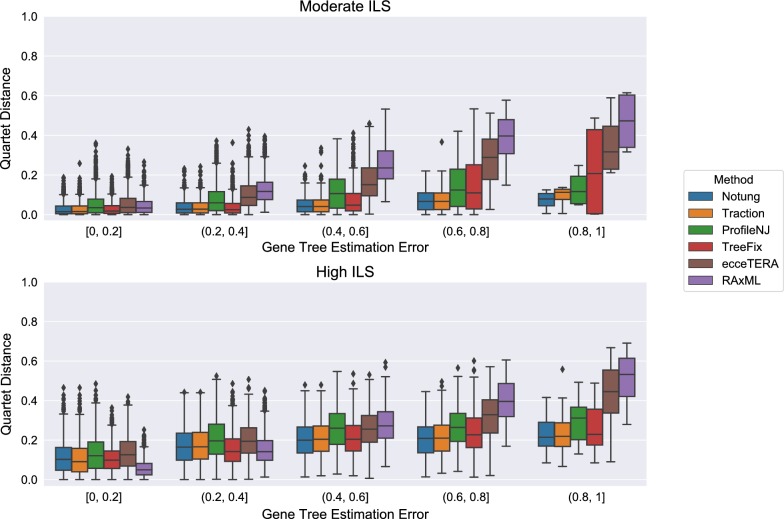
Quartet distance error rates of methods on the ILS-only datasets as a function of GTEE. Results are only shown for those datasets on which all methods completed. Each model condition (characterized by ILS level) has 20 replicate datasets, each with 200 genes

Not all methods completed on all datasets: ecceTERA failed to complete on 67 gene trees, ProfileNJ failed to complete on two gene trees, and all other methods completed on all gene trees. Results shown in Fig. 4 are restricted to those datasets on which all methods completed. For the moderate ILS condition with accuracy evaluated using RF distance (Fig. 4top), all methods were able to improve on RAxML, and the degree of improvement increased with GTEE. For the high ILS condition (Fig. 4bottom), methods improved on RAxML only when GTEE was at least 20%. Thus, GTEE and ILS level both impacted whether methods improved on RAxML. Furthermore, the methods grouped into two sets: TRACTION, Notung, and TreeFix performing very similarly and ProfileNJ and ecceTERA having somewhat higher error. We found the relative performance of these methods follows the same trends for matching (Fig. 5) and quartet distances (Fig. 6) as for RF distances.

### Experiment 2: Comparison of methods on the HGT + ILS datasets

**Fig. 7:**
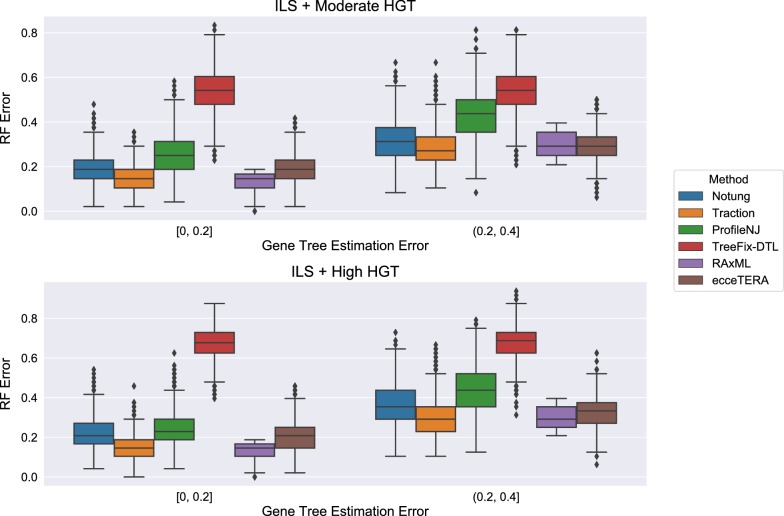
Robinson−Foulds (RF) error rates for ecceTERA as a function of GTEE on ILS + HGT datasets on which it completes. We only show those GTEE conditions for which ecceTERA completed on all genes

The HGT + ILS datasets have heterogeneity due to both HGT and ILS, with the degree of HGT varying from moderate (m5) to high (m6). Here, ecceTERA failed on 1318 datasets with the failure rates increasing as the gene tree estimation error (GTEE) of the initial RAxML gene tree increased: ecceTERA failed 0% of the time when GTEE was less than 40%, 0.4% of the time when GTEE was 40–60%, 23.6% of the time when GTEE was 60–80%, and 90.8% of the time when GTEE was at least 80%. Because of the high failure rate, we report results for ecceTERA on datasets with GTEE of at most 40%; above this level, ecceTERA fails frequently, making comparisons between methods potentially biased. Figure 7 shows that ecceTERA performed well, though not as well as Notung and TRACTION, on these low GTEE datasets.

**Fig. 8:**
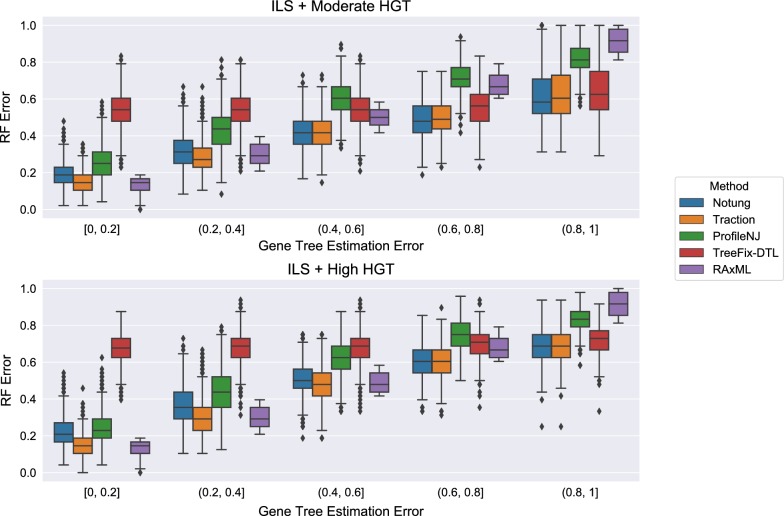
Robinson−Foulds (RF) error rates methods on ILS + HGT datasets as a function of GTEE. Each boxplot displays the distribution of RF error across all replicates for a given method and level of GTEE; ecceTERA is not shown due to a high failure rate on these data

**Fig. 9:**
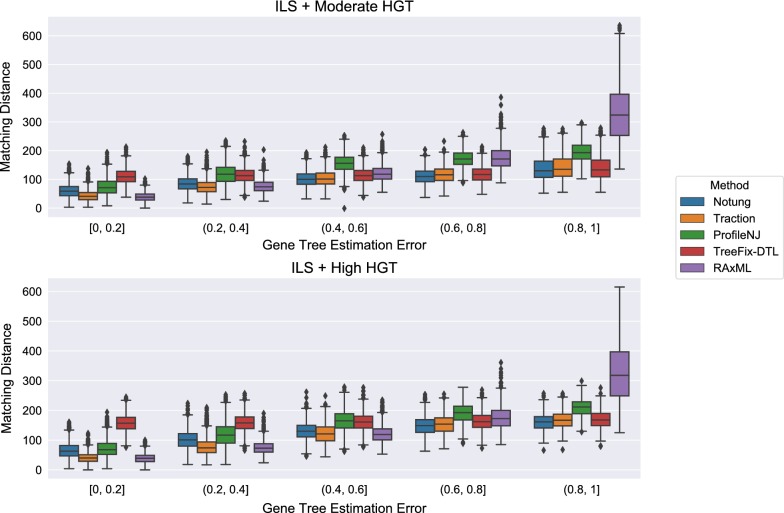
Matching distance error of methods on ILS+HGT datasets as a function of GTEE. Boxplots show a comparison of methods; ecceTERA is not shown due to a high failure rate on these data

**Fig. 10:**
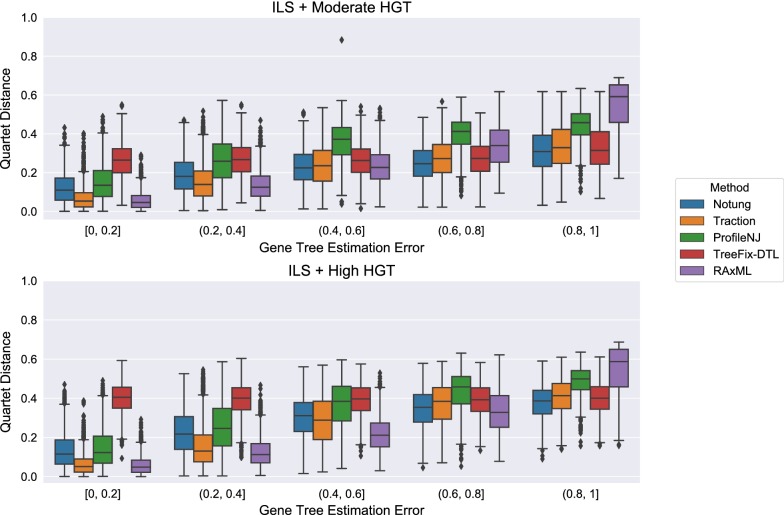
Quartet distance error rates of methods on ILS+HGT datasets as a function of GTEE. Boxplots show a comparison of methods; ecceTERA is not shown due to a high failure rate on these data

Figure 8 shows the impact of the remaining methods on RAxML gene trees as a function of GTEE as measured by RF distance. Figs. 9 and 10 measure this impact using matching distance and quartet distance, respectively. The relative performance between the remaining methods across all evaluation metrics show that TRACTION and Notung were more accurate than ProfileNJ and TreeFix-DTL, with the gap between the two groups increasing with GTEE. We also see that TRACTION had an advantage over Notung for the low GTEE condition and matched the accuracy on the higher GTEE conditions. Finally, for the lowest GTEE bin, no method improved the RAxML gene tree, some methods made the gene trees much less accurate (e.g., ProfileNJ), and only TRACTION maintained the accuracy of the RAxML gene tree. Overall, on the HGT + ILS datasets, TRACTION consistently performed well and provided a clear advantage over the other methods in terms of accuracy.

### Experiment 3: Varying collapse threshold and reference tree on the ILS datasets

**Fig. 11:**
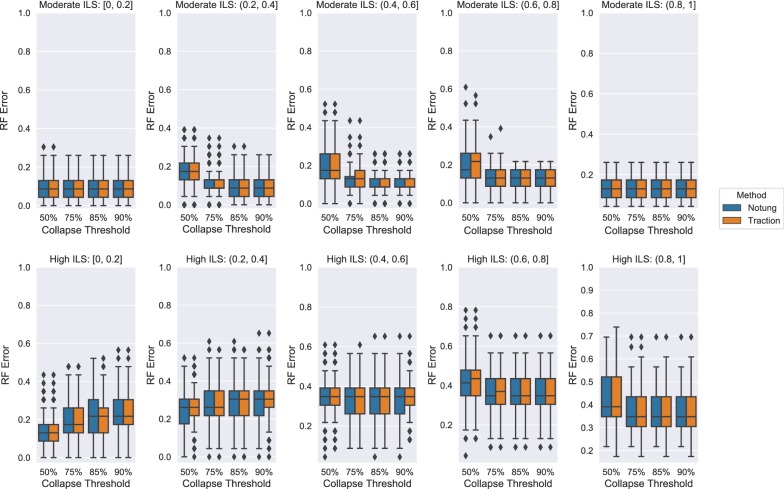
TRACTION and Notung achieve similar RF error rates across collapse thresholds for ILS-only datasets. In each case, edges with support less than the threshold are collapsed before refinement. TRACTION and Notung completed in all instances, so no gene trees are removed

The collapse threshold is an important hyperparameter that may impact the accuracy of gene tree correction methods. We evaluated the effect of this parameter on the two best performing methods from the previous experiments: TRACTION and Notung. Figure 11 shows the results on the ILS-only datasets, stratified by GTEE. Overall, TRACTION and Notung exhibited similar relative performance. Intuitively, increasing the collapse threshold (i.e., collapsing more branches) tends to reduce the error in the moderate ILS condition across all levels of GTEE as well the high ILS condition with sufficiently high GTEE. However, a lower threshold (i.e., collapsing fewer branches) improves accuracy for the low GTEE and high ILS condition, where the original gene tree is well-estimated and the reference species tree is more distant from the true gene trees.

**Fig. 12:**
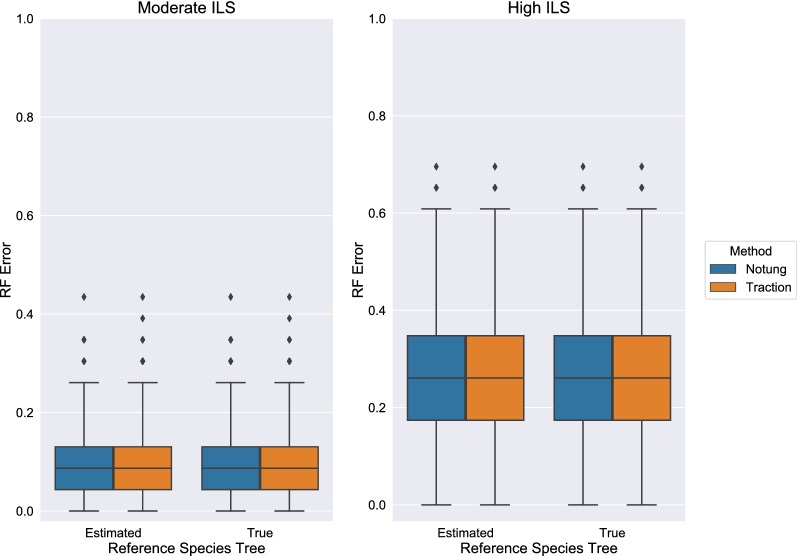
TRACTION and Notung achieve similar RF error rates when using a true species tree as reference. Comparison of using a species tree estimated by ASTRID compared to the true species tree as a reference for gene trees on the ILS-only datasets. TRACTION and Notung completed in all instances, so no gene trees are removed

The reference tree is also an important input that in practice will often itself be estimated. In Fig. 12, we found that using the true model species tree achieves similar absolute performance as using the estimated ASTRID tree as reference. Again, TRACTION and Notung had performed similarly with respect to the RF distance between the true and the estimated (and then corrected) gene tree.

### Running times

We selected a random sample of the 51-taxon HGT + ILS datasets to evaluate the running time (see Table 2). From fastest to slowest, the average running times were 0.5 s for TRACTION, 0.8 s for Notung, 1.7 s for ProfileNJ, 3.8 s for TreeFix-DTL, and 29 s for ecceTERA. Most of the methods had consistent running times from one gene to another, but ecceTERA had high variability, depending on the size of the largest polytomy. When the largest polytomy was relatively small, it completed in just a few seconds, but it took close to a minute when the largest polytomy had a size at the limit of 12. Results on other HGT + ILS replicates and model conditions gave very similar results.

**Table 2:** Total time (in s) for each method to correct 50 gene trees with 51 species on one replicate (label 01) of the HGT + ILS dataset with moderate HGT and sequences of length 100 bp

Method	Time (s)
EcceTERA	1470
NOTUNG	43
TRACTION	30
ProfileNJ	87
TreeFix-DTL	188

### Overall comments

This simulation study shows that the better methods for gene tree correction (TRACTION, Notung, and TreeFix) produced more accurate gene trees than the initial RAxML gene trees for the ILS-only conditions (except for cases where the initial gene tree was already very accurate), and that the improvement could be very large when the initial gene trees were poorly estimated. However, the impact of gene tree correction was reduced for the HGT + ILS scenarios, where improvement over the initial gene tree was only obtained when GTEE is fairly high. As shown in Table 1, the average normalized RF distance between the reference tree (ASTRID) and the true gene trees was never more than 33% for the ILS-only scenarios but very high for the HGT + ILS scenarios (54% for moderate HGT and 68% for high HGT). Since a reference tree (i.e., an estimated species tree) was the basis for the correction of the gene trees, it is not surprising that improvements in accuracy were difficult to obtain for the HGT + ILS scenario. On the other hand, given the large distance between the true species tree and the true gene tree, the fact that improvements were obtained for several methods (TRACTION, Notung, and TreeFix-DTL) is encouraging.

## Conclusions

We presented TRACTION, a method that solves the RF-OTRC problem exactly in $O(n^{1.5}\log n)$ time, where *n* is the number of species in the species tree; the algorithm itself is very simple, but the proof of optimality is non-trivial. TRACTION performs well on singly-labeled gene trees, matching or improving on the accuracy of competing methods on the ILS-only datasets and dominating the other methods on the HGT + ILS datasets. Furthermore, although all the methods are reasonably fast on these datasets, TRACTION is the fastest on the 51-taxon gene trees, with Notung a close second.

The observation that TRACTION performs as well (or better) than the competing methods (ecceTERA, ProfileNJ, Notung, TreeFix, and TreeFix-DTL) on singly-labeled gene trees under ILS and HGT is encouraging. However, the competing methods are all based on stochastic models of gene evolution that are inherently derived from gene duplication and loss (GDL) scenarios (and in one case also allowing for HGT), and thus it is not surprising that GDL-based methods do not provide the best accuracy on the ILS-only or HGT + ILS model conditions we explore (and to our knowledge, all the current methods for gene tree correction are based on GDL models). Yet, TRACTION has good accuracy under a wide range of scenarios for singly-labeled gene trees. We conjecture that this generally good performance is the result of its non-parametric criterion which can help it to be robust to model mis-specification (of which gene tree estimation error is one aspect).

This study shows that when the reference tree is very far from the true gene trees (e.g., our HGT + ILS data), gene tree correction typically fails to improve the initial gene tree and some methods can make the gene tree worse. This brings into question why the species tree (whether true or estimated) is used as a reference tree. We note that while the GDL-based methods may benefit from the use of a species tree as a reference tree (since the correction is based on GDL scenarios), this type of reference tree may not be optimal for TRACTION, which has no such dependency. Thus, part of our future work will be to explore techniques (such as statistical binning [37, 38]) that might enable the estimation of a better reference tree for TRACTION in the context of a multi-locus phylogenomic analysis.

This study suggests several other directions for future research. The GDL-based methods have variants that may enable them to provide better accuracy (e.g., alternative techniques for rooting the gene trees, selecting duplication/loss parameter values, etc.), and future work should explore these variants. Most gene tree correction methods have been developed specifically to address the case where genes have multiple copies of species as a result of gene duplication events. We showed that a naive extension of TRACTION to handle multi-labeled genes by using a generalization of the RF distance based on an extended species tree, such as proposed in [18], can lead to misleading results. Future work should explore other generalizations of RF distance that do not suffer from these same limitations, and consider other distances between MUL-trees, as discussed in [39]. Recent work has shown how Notung could be extended to address HGT [40]; a comparison between TRACTION and a new version of Notung that addresses HGT will need to be made when Notung is modified to handle HGT (that capability is not yet available). Finally, the effect of gene tree correction on downstream analyses should be evaluated carefully.

## Data Availability

TRACTION is available at [41] and the study datasets are available at [42].
